# *RORC2* Genetic Variants and Serum Levels in Patients with Rheumatoid Arthritis

**DOI:** 10.3390/ijms17040488

**Published:** 2016-04-01

**Authors:** Agnieszka Paradowska-Gorycka, Barbara Stypinska, Andrzej Pawlik, Katarzyna Romanowska-Prochnicka, Ewa Haladyj, Malgorzata Manczak, Marzena Olesinska

**Affiliations:** 1 Department of Biochemistry and Molecular Biology, National Institute of Geriatrics, Rheumatology and Rehabilitation, Spartańska 1, 02-637 Warsaw, Poland; barbara.stypinska@wp.pl; 2 Department of Pharmacology, Pomeranian Medical University, 70-111 Szczecin, Poland; pawand@poczta.onet.pl; 3 Department of Connective Tissue Diseases, National Institute of Geriatrics, Rheumatology and Rehabilitation, 02-637 Warsaw, Poland; katarzyna.prochnicka@gmail.com (K.R.-P.); ehaladyj@o2.pl (E.H.); marzena.olesinska@vp.pl (M.O.); 4 Department of Epidemiology and Health Promotion, National Institute of Geriatrics, Rheumatology and Rehabilitation, 02-637 Warsaw, Poland; m.manczak@op.pl

**Keywords:** Th17 cells, rheumatoid arthritis, pathogenesis, RORc, polymorphisms

## Abstract

Background: In the present study, we aimed to evaluate whether polymorphisms within the *RORc2* gene are involved in the risk and severity of rheumatoid arthritis (RA). Methods: 591 RA patients and 341 healthy individuals were examined for *RORc2* gene polymorphisms. Serum retinoic acid receptor-related orphan receptor C (RORc) levels were measured by enzyme-linked immunosorbent assay (ELISA). Results: The rs9826 A/G, rs12045886 T/C and rs9017 G/A *RORc2* gene SNPs show no significant differences in the proportion of cases and control. Overall, rs9826 and rs9017 were in high linkage disequilibrium (LD) with D’ = 0.952 and *r*^2^ = 0.874, except rs9826 and rs12045886; and rs12045886 and rs9017 in weak LD. The genotype–phenotype analysis showed a significant association between *RORc2* rs9826 A/G and rs9017 G/A single nucleotide polymorphisms (SNPs) and median of C-reactive protein (CRP). Serum RORc levels was higher in RA patients with rs9826AA, rs12045886TT and -TC, and rs9017AA genotypes compared to healthy subjects with the same genotypes (*p* = 0.02, *p* = 0.04 and *p* = 0.01, respectively). Moreover, the median of RORc protein level was higher in RA patients with number of swollen joints bigger then 3 (*p* = 0.04) and with Health Assessment Questionnaires (HAQ) score bigger then 1.5 (0.049). Conclusions: Current findings indicated that the *RORc2* genetic polymorphism and the RORc2 protein level may be associated with severity of RA in the Polish population.

## 1. Introduction

Since increasing interleukin (IL)-17 concentrations were observed in autoimmune diseases, increased attention has led to a precise definition of the role of human Th17 cells and their products in the process of ongoing immune response involved tissues [1,2,3]. The role of Th17 cells and Th17-associated cytokines in the pathogenesis of rheumatoid arthritis (RA) is now widely recognized. Th17 cells, which are the dominant effector T cell involved in the induction of autoimmune chronic tissue by promoting the immune response, are much more effective in inducing inflammation than any other Th1 cell recognized thus far and is therefore the “main culprit” of autoimmune diseases [4]. Th17 cell produce several cytokines including IL-17, IL-17F, IL-6, IL-21, IL-22 and tumor necrosis factor-alpha (TNF-α) that play a key role not only in the RA but also in the pathogenesis of experimental autoimmune encephalomyelitis (EAE) and collagen induced arthritis (CIA), and they induces dendritic cells (DCs) to produce IL-12 and interferon (IFN-c) [5,6,7,8,9,10]. Th17 cell present in the joint may create a positive feedback loop leading to the continuous activation of T cells, which is a critical event in the generation of autoimmunity [11].

Although Th17 cells express numerous markers, human *RORc2* (nuclear hormone retinoic acid receptor-related orphan receptor variant 2; a short isoform of *RORc* gene, encoded by the human *RORc* gene located on chromosome 1q21–q23) ortholog of mice *RORγt*, is a master transcriptional factor that can drive Th17 differentiation in both humans and mouse, respectively [12,13,14,15,16]. Overexpression of *RORc2* by inducing IL-17, IL-26, TCR and CCR6 stimulates a wide range of phenotypic and functional programming during Th17 cells differentiation [17,18]. Knockdown of transcription factor RORc cause high Foxp3 levels and reduces expression of pro-inflammatory cytokines such as IL-1β, IL-6, IL-17, IFN-γ and TNF-α, suggesting that the role of *RORc2* in Th17 cells differentiation involves not only in induction of Th17 characteristics genes, but also suppression of Treg cells specific programs [18,19,20].

The research of the Th17 cells development may help better understand its role in the pathogenesis of RA. However, there was still no too much study in this field. In this study, we hypothesized that the *RORc2* gene is responsible for Th17 cells and IL-17-producing Treg cells differentiation may also be a strong molecular candidate for rheumatoid arthritis severity and/or susceptibility. To test this hypothesis, we examined, for the first time, three candidate single nucleotide polymorphisms (SNPs) in the *RORc2* gene, rs9826 A/G, rs12045886 T/C and rs9017 G/A, and RORc protein expression and determined their possible association with susceptibility to and clinical phenotype of RA in Polish population.

## 2. Results

### 2.1. RORc2 SNPs Information and Association of the Individual SNPs with Risk of Rheumatoid Arthritis (RA)

**Figure 1:**
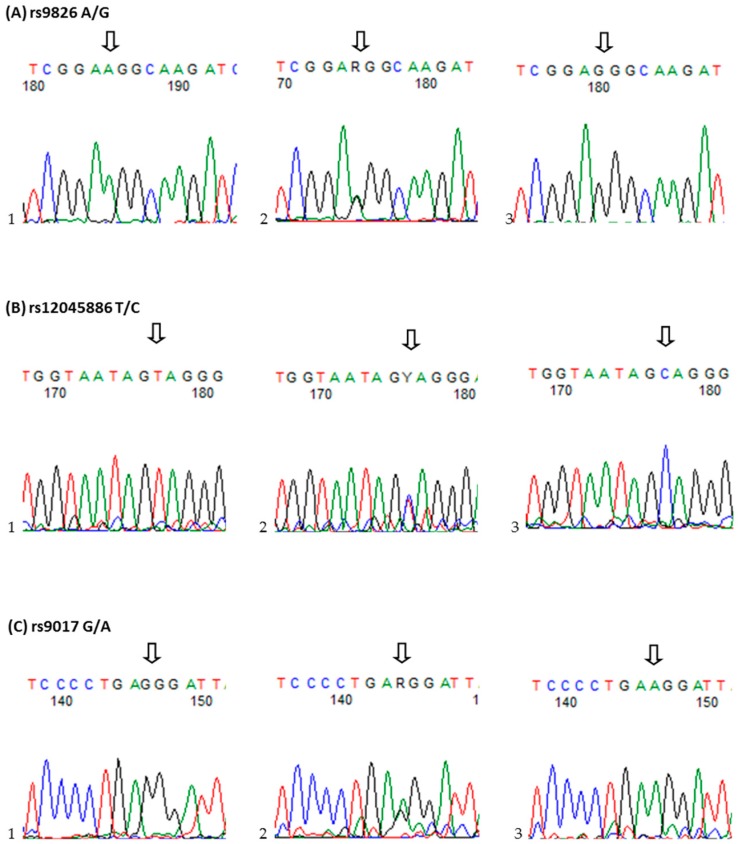
Sequencing map of genotype for *RORc2* gene. (**A**) rs9826 A/G, the arrow of 1–3 showed AA, AG and GG genotypes, respectively.; (**B**) rs12045886 T/C, the arrow of 1–3 showed TT, TC and TT genotypes, respectively; (**C**) rs9017 G/A, the arrow of 1–3 showed GG, GA and AA genotypes, respectively.

To confirm the genotyping results, PCR-amplified randomly selected DNA samples were analyzed on ABI PRISM Sequencer (Applied Biosystems, Foster City, CA, USA), and the results were 100% concordant (Figure 1). The *RORc2* rs9826 A/G, rs12045886 T/C and rs9017 G/A polymorphism genotype distribution were in Hardy-Weinberg equilibrium (HWE) in both patients and control group (Table 1). Moreover, there was no evidence of any systematic bias in genotyping. The MAF of the three SNPs in our samples were similar to those in the Utah residents of northern and western European ancestry (HapMap database; Table 1).

The genotyping success was greater than 87% in all cases. The distributions of genotype and allele frequencies of the polymorphisms rs9826 A/G, rs12045886 T/C and rs9017 G/A in *RORc2* among patients and controls, as well as their associations with the risk of RA were shown in Table 2. Three genetic models, including codominant, dominant and recessive were applied to assess the association of SNPs within the *RORc2* gene and RA risk. There were no significant differences in the proportion of cases and control under each genetic model for tested polymorphisms. Effect sizes were adjusted for sex and age and results were still insignificant.

### 2.2. RORc2 Haplotype Analysis and Risk of Rheumatoid Arthritis

**Figure 2:**
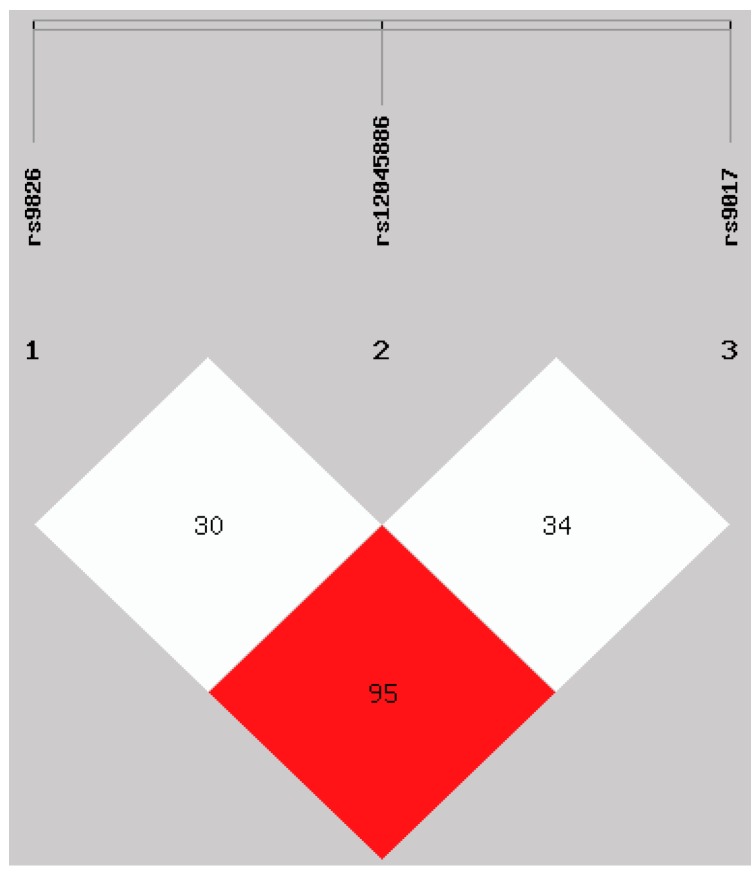
Linkage disequilibrium (LD) plots of three SNPs in the *RORc2* gene. The plot illustrates pairwise LD between all polymorphisms based on D′ values. Values approaching zero indicate absence of LD, and those approaching 100 indicate complete LD. The square colored red represent varying degrees of LD < 1 and LOD (logarithm of odds) > 2 scores (strong LD) and white blocks represent varying degrees of LD < 1 and LOD < 2 scores (weak LD).

Next, we evaluated the interaction between examined *RORc2* gene polymorphisms and their inheritance by analyzing the distribution of haplotypes in RA patients and control group. The interaction between any possible pair of SNPs was visualized by SHEsis program (Figure 2). Analysis revealed high linkage disequilibrium (LD) between rs9826 and rs9017 (D’ = 0.952 and *r*^2^ = 0.874), but weak LD between rs9826 and rs12045886; and rs12045886 and rs9017 (D’ = 0.309 and *r*^2^ = 0.059; D’ = 0.345 and *r*^2^ = 0.070, respectively). The eight potential haplotypes were formed, as present in Table 3. Four major *RORc2* haplotypes (ACA, ATA, GCG, and GTG), with frequency >5%, were observed in RA patients as well as in healthy subjects. Among them, the most frequent haplotype was ATA, which was estimate on 589 of the 1186 chromosome with a frequency of 49.6% in RA patients and on 367 of the 682 chromosomes with frequency of 53.8% in control group. This ATA haplotype demonstrated significantly lower frequencies in RA patients compared to controls (*p* = 0.04, odds ratio (OR) = 0.820, confidence interval (CI) = 0.677–0.994). This association is translated into a protective effect. There were no differences in haplotype frequency between RA patients and control group for the other haplotypes.

### 2.3. The Association of the RORc2 rs9826 A/G, rs12045886 T/C and rs9017 G/A Polymorphisms with RA Phenotype

Although we found no association between examined *RORc2* and susceptibility to RA, we analyzed the potential association between both the *RORc2* gene SNPs and laboratory and disease activity parameters in our RA group. We performed a stratified analysis of combined genotypes under the dominant and the recessive models for each examined polymorphisms.

As reported, without Bonferroni correction the genotype-phenotype analysis showed significant differences in the disease duration of RA, median value of CRP, VAS score, and creatinine level in the *RORc2* rs9826 A/G polymorphisms under recessive model ((AA + AG) *vs.* GG). The disease duration was higher in RA patients with rs9826 GG genotype (*p* = 0.02), whereas CRP, VAS and creatinine level were higher in RA patients with combined genotype rs9826 AA + AG (*p* = 0.001, *p* = 0.01, *p* = 0.02, respectively). However, after Bonferroni correction for multiple testing there was an association only between *RORc2* rs9826 A/G SNP and median value of CRP (Table 4). Under the dominant model, without and after Bonferroni correction (Table S1 in Supplementary Materials), there were no significant associations between *RORc2* rs9826 A/G polymorphisms and RA phenotype.

The influence of *RORc2* rs12045886 T/C polymorphism on clinical symptoms of RA without Bonfferoni correction showed significant differences in number of women under dominant model (Table S2 in Supplementary Materials) and with median value of PLT under the recessive model (Table S3 in Supplementary Materials). Median value of PLT was higher and number of women was lower in RA patients with combined genotype rs12045886 TT + TC in comparison to RA patients with CC genotype (*p* = 0.02 and *p* = 0.006, respectively). After Bonferroni correction there were no significant associations between SNP and RA phenotype.

The analysis of the third *RORc2* gene polymorphism at position rs9017 G/A, without Bonferroni correction, showed that the duration of RA and creatinine level were significantly higher in carriers of the GG genotype in comparison with rs9017 GA + AA subjects. In contrast, carriers of the combined rs9017 GA + AA genotype were a higher median value of CRP, VAS, HAQ and DSA-28 score. However, after Bonferroni correction we found no association between *RORc2* rs9017 G/A SNP and RA phenotype under the recessive models (Table S4 in Supplementary Materials). Furthermore, under the dominant model, after Bonferroni correction, we observed correlation only between *RORc2* rs9017 G/A polymorphisms and median value of CRP (*p* = 0.0005). Other associations were no significant (Table 5).

### 2.4. RORc2 Protein Levels in RA Patients and Controls in Relation to RA Clinical Parameters

**Figure 3:**
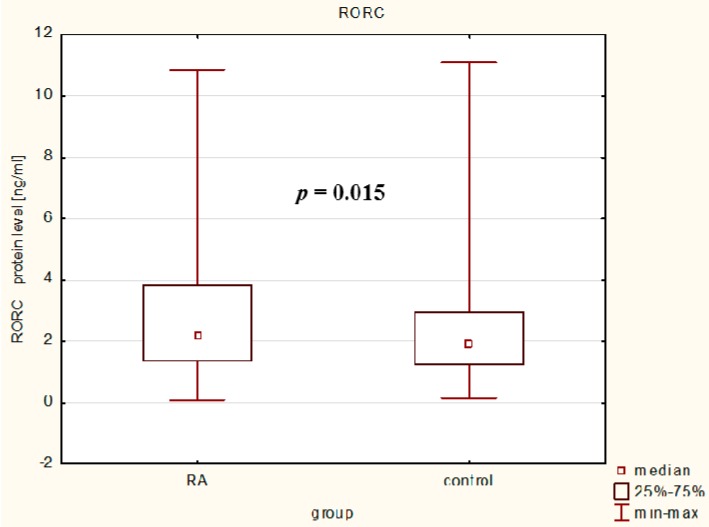
RORc2 protein level in RA patients and healthy subjects.

We analyzed the *RORc* expression level in serum samples from 278 RA patients and 295 healthy individuals. As shown in Table S5 in Supplementary Materials and Figure 3 a significantly higher RORc protein expression level (median (IQR): 2.2 (1.4–3.8)) was found in RA patients compared with that in healthy individuals (median (IQR): 1.9 (1.4–3.0); *p* = 0.02). We next conducted a comparative analysis between median value of RORc protein serum levels and clinical parameters of RA patients (Table 6). We found the median value of RORc protein level was higher in RA patients with number of swollen joints bigger then 3 (*p* = 0.04) and with HAQ score bigger then 1.5 (0.049). Moreover, we also observed that in RA patients with higher RORc serum protein level were a tendency to a higher median value of CRP and DAS-28 score comparing with RA patients with lover RORc protein levels.

### 2.5. RORc2 Gene Polymorphisms with Respect to Serum RORc Levels

To study the relation between genotype and phenotype, we investigated also RORc protein serum levels in RA patients and controls with respect to *RORc2* gene polymorphisms.

First, we performed an analysis between patients and controls according to rs9826 A/G, rs12045886 T/C and rs9017 G/A *RORc2* genotypes (Table 7). Serum levels of RORc in RA patients with rs9826 AA, rs12045886 TC and rs9017 AA genotypes were significantly higher than healthy subjects with the same *RORc2* genotypes (*p* = 0.02, *p* = 0.04 and *p* = 0.01, respectively). Tendency to increased serum levels of RORc was also observed in RA patients with rs12045886 TT genotype compered to controls with the same genotypes (*p* = 0.09).

**Figure 4:**
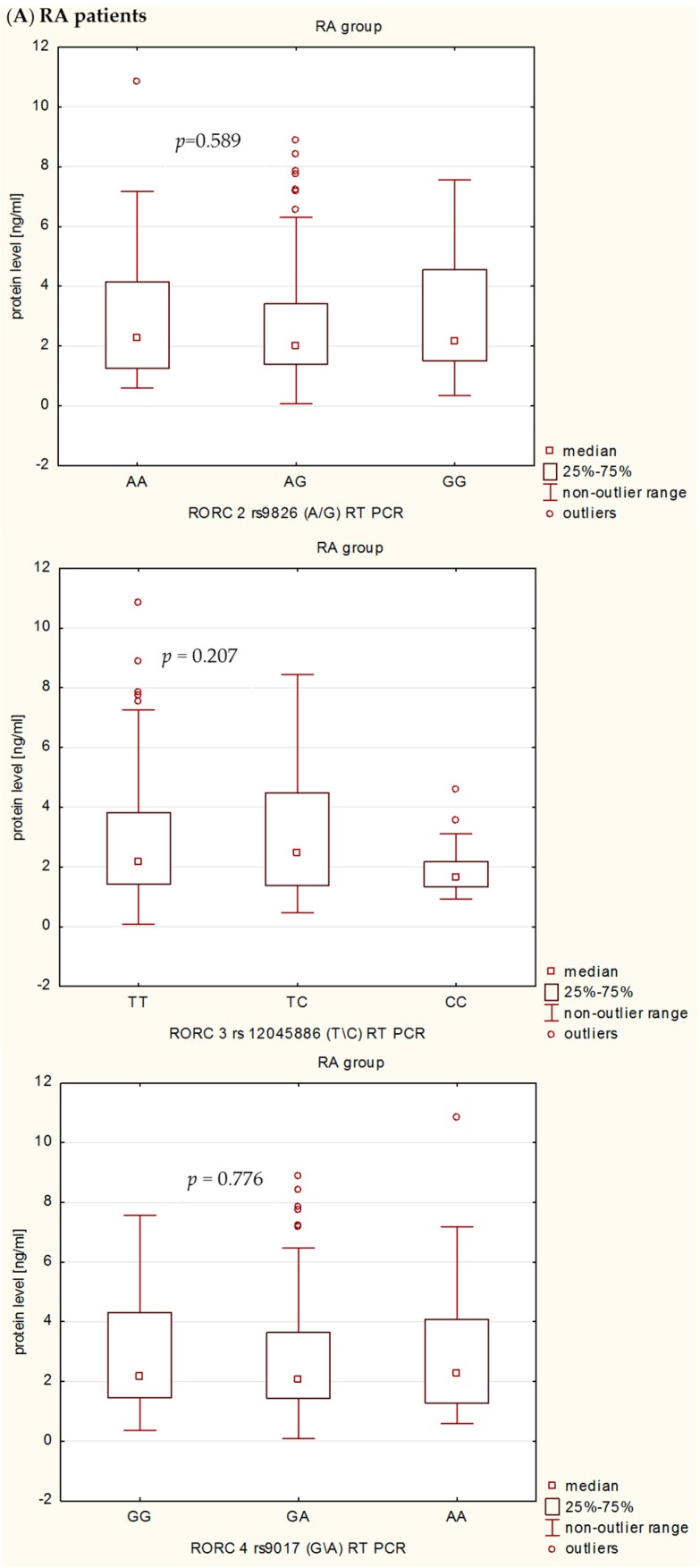

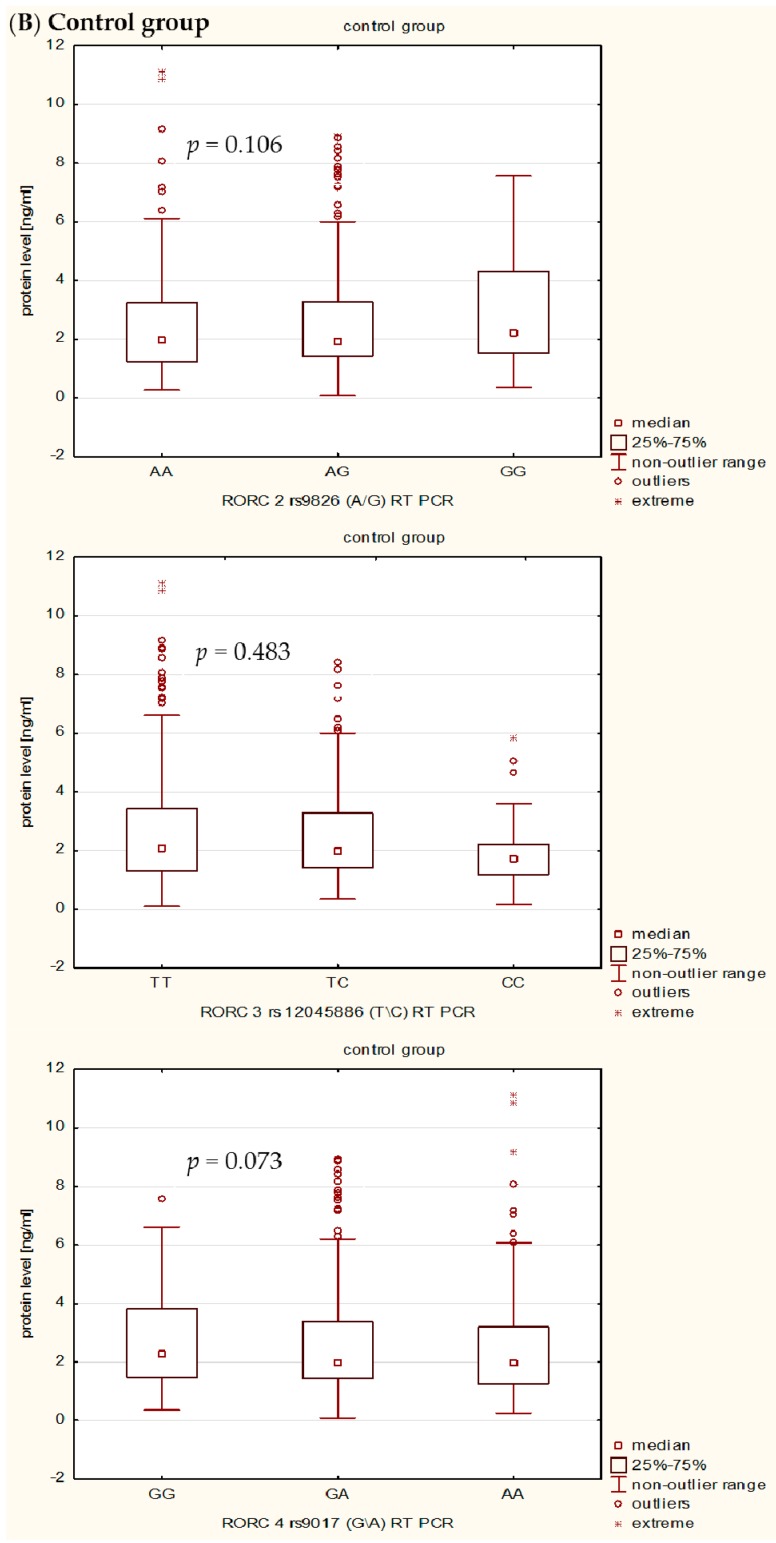
Variation in RORc expression levels in (**A**) RA patients and (**B**) control group in relation to *RORc2* genotypes: (**A**) rs9826 A/G, *p* = 0.589; rs12045886 T/C, *p* = 0.207; and rs9017 G/A, *p* = 0.776; and (**B**) rs9826 A/G, *p* = 0.106; rs12045886 T/C, *p* = 0.483; and rs9017 G/A, *p* = 0.073. *p*, Kruskal–Wallis test; *p* < 0.05 was considered significant.

We next examined the correlation between serum RORc levels in RA patients and healthy subjects in relation to rs9826 A/G, rs12045886 T/C and rs9017 G/A *RORc2* genotypes (Figure 4). In this case, we find no differences, neither among RA patients nor in healthy subjects. Only in control group in relation to the *RORc2* rs9017 G/A polymorphism we observed a tendency to the lower serum RORc levels in individuals with AA genotype than in subject with GG or AG genotypes. Moreover, in RA patients with rs9017 AA the serum RORc levels were higher than in RA patients with rs9017 GG or GA genotypes, although this association was not significant.

## 3. Discussion

Over the last decade, numerous studies have disclosed that both Th1 cells as well as Th17 cells are involved in the pathogenesis of RA, where higher frequency of Th17 cells had been found in the arthritic synovium [21,22]. In addition to RA, it has been presented that Th17 cells are also involved in the pathogenesis of other autoimmune diseases through the promotion of tissue inflammation, the mobilization of the innate or adaptive immune system and secretion of the pro-inflammatory cytokines and chemokines [21,23,24]. Because RORc2 function as a lineage-specifying transcriptional factor of Th17 cells and play a key role in development and maintenance of these cells [12,19,25,26], *RORc2* gene may represent a candidate gene for autoimmune diseases. However, not too much is known about the function of *RORc2* genetic variants in autoimmune diseases, including RA. The *RORc2* gene polymorphisms have been analysis only in a Behcet’s disease (BD) [27], type 2 diabetes (T2DM) [15] and secondary lymphedema [27]. This is why analysis of polymorphisms within the *RORc2* gene may help to uncover their correlations with some biochemical and laboratory findings. Liao D. *et al.* [27] in the sole report exploring *RORc* genetic variants in BD studied the 25 selected taqSNPs and copy number variants (CNVs) in region of *RORc* gene. They concluded that the high RORc CNV was associated with susceptibility to BD and that the RORc mRNA expression levels were increased in patients with high *RORc* copy number. The study of Wang H. *et al.* [15] demonstrated that from 11 examined SNPs, in African American individuals and in Caucasian individuals from northern European, only the Ala454Gly variant was three-fold more common among African American patients with T2DM than in healthy subjects. Furthermore, Newman B. *et al.* [20] found that *RORc* tagSNP rs11801866 located at the 5′ end of *RORc* gene was associated with susceptibility to secondary lymphedema and together with rs12128071 are predicted to affect transcription factor binding sites.

In our study, we observed that serum RORc levels were higher in RA patients than in controls reflecting the ongoing inflammatory process in rheumatoid arthritis and it confirms and extends the hypothesis that Th17 enrichment occurs in inflamed joints [13]. Some previous reports have presented the RORc mRNA expression [13,18,20,23,28], but our study is the first that exploring the RORc protein levels in the serum of RA patients. Our results indicate that RA patients with higher median value of RORc protein levels have higher disease activity and in our opinion, probably an increased number of Th17 cells than RA patients with lower median value of RORc protein levels and controls. As shown in earlier studies, active RA patients had a significantly higher frequency of peripheral Th17 cells and RORc mRNA expression than inactive RA patients and healthy subjects suggesting a potential predominant role of Th17 cells in the inflammatory process and chronic progression in RA [21,29]. We conclude that by induction of Th17-characteristics genes, suppression of regulatory T cell-specific program and controlling pro-inflammatory cytokines/chemokines expression, RORc might play an essential role in the regulation of inflammatory processes and therefore would represent an innovative therapeutic regiment for the balance between immunity and tolerance.

Considering an important role of the RORc2 in the development of the Th17/Treg cells and differences in genetic predispositions between populations [13,18,19,30], we decided to carry out an analysis of selected *RORc2* genetic variants in relation to RA. To the best our knowledge, this study is the first in which the associations between not only serum RORc levels, but also *RORc2* gene SNPs and haplotypes have been investigated in a cohort of patients with rheumatoid arthritis. For the analysis we chose the rs9826 A/G and rs9017 G/A polymorphisms located in the 3′UTR, and rs12045886 T/C polymorphism located in an intronic region. SNPs located in the 3′UTR region of the *RORc2* gene may interfere with mRNA translation and stability through effects on polyadenylation and regulatory miRNA-mRNA and protein-mRNA interactions, thus affecting the level of protein expression. However, this needs to be verified in further studies.

Interestingly, when we studied each of the polymorphisms separately, our results have shown no association with the susceptibility to RA in Polish population. A relatively small population size might have prevented from seeing the association. The proportion of genetic contribution of certain polymorphic locus to RA susceptibility may be influenced by other local environmental and/or joint-specific genetic factors most of which were postulated to be involved in cell signaling. Moreover, when we examined all the polymorphisms to create haplotypes, our study showed that a protective effect of the ATA haplotype against the risk of rheumatoid arthritis in our populations, suggesting that the impact of gene on disease risk may not limit to single SNP. Additionally, the results derived from our study are the first, which showing haplotype-based association analysis for these three genetic variants in the *RORc2* gene.

In the present study, we also investigated the influence of rs9826 A/G, rs12045886 T/C and rs9017 G/A *RORc2* gene polymorphisms on clinical phenotype and RORc serum levels in RA patients. Our detailed genotype-phenotype analysis indicated that without Bonferroni correction: (i) the *RORc2* rs9826 A/G polymorphisms under recessive model ((AA + AG) *vs.* GG) was associated with the disease duration of RA, median value of CRP, VAS score, and creatinine level; (ii) the rs12045886 T variant was associated with a significantly higher median value of PLT and lower number of women; and (iii) the carriers of the combined rs9017 GA + AA genotype were a higher median value of CRP, VAS, HAQ and DSA-28 score. However, after Bonferroni correction, there was an association only between *RORc2* rs9826 A/G and rs9017 G/A SNPs and median value of CRP. In addition, we can also observed that RA patients with the *RORc2* rs9017A allele had more advanced disease than rs9017 G allele carriers, suggesting that this polymorphism might be associated with higher disease activity. We also found evidence for association of the *RORc2* rs9826 A/G, rs12045886 T/C and rs9017 G/A polymorphisms with difference in RORc serum levels between RA patients and controls. RORc serum levels were significantly higher in RA patients with rs9826AA, rs12045886TT and TC, and rs9017AA genotypes compared to healthy subjects with the same genotypes. Moreover, RA patients with rs9017AA genotype had a tendency to higher RORc levels in serum than RA patients with rs9017GG or GA genotypes. The expression level of the transcriptional factors within the target cells is a reflection of the transcriptional processes, whereas in the serum reflects passively the undergoing regulation. Moreover, we believe that analysis of transcriptional factors level in the serum may reflect pathological processes such as cell damage. In our opinion, RORc might increase with disease progression as their serum levels would depend much more on the number and activity of Th17 cells, which are typical pro-inflammatory cells promoting the induction of autoimmune tissue inflammation and plays a key role in the development of autoimmune arthritis, than on the sole genotype. Despite this observation, we infer that the analyzed polymorphisms might still affect RORc2 mRNA and protein intracellular expression. However, further studies with larger sample size will be needed to validate the genetic effects of the *RORc2* gene polymorphisms on RA. Moreover, we believe that our results could be helpful to clarify the role of RORc in the pathogenesis of RA, which it might be a candidate factor consistent with the severity of disease.

## 4. Materials and Methods

### 4.1. Study Participants

In total, 591 patients with established RA and 341 unrelated healthy controls were included in this study. Blood samples were obtained from the patients recruited from the National Institute of Geriatrics, Rheumatology and Rehabilitation in Warsaw and Pomeranian Medical University in Szczecin, Poland. All patients had a diagnosis of RA, and met the RA classification criteria published by the American College of Rheumatology in 1987. Clinical and biochemical data were collected from patient’s files and questionnaires after signed informed consent and are presented in Table 8.

The control group (217 females and 124 males, age between 18 and 63 years) consisted of healthy volunteers who showed no clinical and/or laboratory signs of any autoimmune disease. They were selected randomly from blood bank donors. Patients and healthy subjects were from the same geographical area and they had the same socioeconomic status. For the present study, we selected a representative sample of the admixed urban Polish population.

The study was reviewed and approved by the Research Ethics Committee of the Institute of Rheumatology and Pomeranian Medical University.

### 4.2. DNA Extraction

Genomic DNA from RA patients and healthy subjects was extracted from whole blood using the standard isothiocynate guanidine (GTC) extraction method and/or the QIAamp DNA Blood Mini Kit (Qiagen, Hilden, Germany).

### 4.3. Single-Nucleotide Polymorphisms Selection and Genotyping

The three *RORc2* (NC_000001.11) SNPs studied, rs9826 A/G, rs12045886 T/C and rs9017 G/A, were selected on the basis of HapMap data release 24/phase II, November 2008, NCBI build 36, dbSNP b126.Genotyping was analyzed using TaqMan SNP genotyping assay (Applied Biosystems, Foster City, CA, USA): C_9624797_20 (rs9826), C_11260074_10 (rs12045886), and C_9624790_20 (rs9017). The reaction was performed in 10 µL volumes on StepOne real-time PCR system in RotorGene 6000 Real-Time PCR system with the fallowing amplification protocol: denaturation at 95 °C for 10 min, followed by 40 cycles of denaturation at 92 °C for 15 s, and annealing and extension at 60 °C for 1 min.

### 4.4. Serum Levels of ROR_C_ Determination

Blood samples were obtained from patients and controls, centrifuged and stored at −86 °C until analysis. Serum RORc levels (ng/mL) were determined by enzyme-linked immunosorbent assay kits (ELISA: Wuhan EIAab Science Co., Ltd., Wuhan, China), according to the manufacturer’s instructions. The minimum level of detection for RORc was 0.15 ng/mL. Plates were read at an absorbance of 450 nm on Elx800 reader (BIO-TEK Instruments, Winooski, VT, USA).

### 4.5. Statistical Analysis

Deviations from Hardy–Weinberg equilibrium expectations were determining using the software available at Institute of Human Genetics, Germany. To evaluate the extent of linkage disequilibrium (LD), D′ and *r*^2^ between pairs of polymorphisms was quantified using the SHEsis software [31,32]. Logistic regression under three genetic models (codominant, dominant and recessive) was used to estimate the odds ratio (OR) and 95% confidence interval (CI) for the risk genotype. A probability value of *p* < 0.017 (according to Bonferroni correction) was considered significant. Normality of the distribution of continuous variables was assessed using the Shapiro–Wilk test. The correlation between genetic variants and phenotype of RA were compared by: (1) Fisher exact test (depending on expected values) or χ^2^ test or χ^2^ test with Yates’ correction for categorical variables; and (2) Mann–Whitney *U* test for continuous variable. Continuous variables are expressed as the median and interquartile range (IQR), whereas categorical variables as number and percentage. Bonferroni correction was used to adjust *p*-values for multiple measures (Bonferroni-corrected α-level = 0.05/16). Statistical significance was assumed at the *p* < 0.003 level.

## Figures and Tables

**Table 1:** SNPs information and genotyping results for rheumatoid arthritis (RA) patients and control group.

SNP ID	Allele	SNP Type	MAF			*p* (HWE)	
			RA	Control	HapMap-CEU	RA	Control
rs9826	A/G	3′UTR	0.38	0.34	0.39	0.12	0.55
rs12045886	T/C	Intron	0.27	0.25	0.33	0.18	0.53
rs9017	G/A	3′UTR	0.64	0.64	0.60	0.14	0.95

MAF, minor allele frequency; HWE, Hardy-Weinberg equilibrium; CEU, Utah residents of northern and western European ancestry.

**Table 2:** Genotype and allele frequencies of the *RORc2* polymorphisms in RA patients and controls.

*RORc2* SNP	Genotype	RA *n* (%)	Controls *n* (%)	Unadjusted OR (95% CI)	*p* Value	Adjusted OR (95% CI)	*p* Value
rs9826 (A/G)							
Codominant	AA	221 (50%)	144 (43%)	1	–	1	–
	AG	295 (37%)	157 (46%)	1.04 (0.86–1.27)	0.68	1.14 (0.83–1.55)	0.42
	GG	75 (13%)	37 (11%)	1.13 (0.85–1.49)	0.41	1.03 (0.66–1.60)	0.89
Dominant	AA	221 (50%)	144 (43%)	1	–	1	–
	AG + GG	370 (63%)	194 (57%)	1.12 (0.97–1.28)	0.12	1.14 (0.92–1.43)	0.23
Recessive	AA + AG	516 (87%)	301 (89%)	1	–	1	–
	GG	75 (13%)	37 (11%)	1.09 (0.88–1.34)	0.43	1.01 (0.73–1.41)	0.93
**rs12045886 (T/C)**							
Codominant	TT	281 (53%)	190 (56%)	1	–	1	–
	TC	221 (41%)	132 (39%)	1.04 (0.81–1.33)	0.75	1.35 (0.92–1.98)	0.12
	CC	32 (6%)	19 (6%)	1.05 (0.71–1.55)	0.82	1.61 (0.34–1.10)	0.10
Dominant	TT	281 (53%)	190 (56%)	1	–	1	–
	TC + CC	253 (47%)	151 (44%)	1.06 (0.93–1.22)	0.37	1.00 (0.80–1.24)	0.98
Recessive	TT + TC	502 (94%)	322 (94%)	1	–	1	–
	CC	32 (6%)	19 (6%)	1.04 (0.78–1.39)	0.80	0.69 (0.44–1.08)	0.10
**rs9017 (G/A)**							
Codominant	GG	67 (13%)	43 (13%)	1	–	1	–
	GA	262 (50%)	157 (46%)	1.09 (0.90–1.33)	0.39	1.17 (0.86–1.60)	0.31
	AA	194 (37%)	141 (41%)	0.90 (0.73–1.10)	0.31	0.94 (0.68–1.30)	0.72
Dominant	GG	67 (13%)	43 (13%)	1	–	1	–
	GA + AA	456 (87%)	298 (87%)	0.99 (0.81–1.22)	0.93	1.09 (0.79–1.49)	0.61
Recessive	GG + GA	329 (63%)	200 (59%)	1	–	1	–
	AA	194 (37%)	141 (41%)	0.92 (0.80–1.05)	0.21	0.92 (0.74–1.15)	0.47

OR, odds ratio; CI, confidence interval; Adjusted OR for sex and age.

**Table 3:** *RORc2* haplotypes in RA patients and controls.

Haplotype rs9826/rs12045886/rs9017	RA *n* = 1186 (%)	Controls *n* = 682 (%)	OR (95% CI)	*p* Value
A C A	133 (11.2)	63 (9.2)	1.235 (0.900–1.695)	0.19
A C G	9 (0.8)	8 (1.2)	–	–
A T A	589 (49.5)	367 (53.8)	0.820 (0.677–0.994)	**0.04**
A T G	11 (0.9)	8 (1.2)	–	–
G C A	2 (0.1)	3 (0.4)	–	–
G C G	172 (14.5)	97 (14.2)	1.016 (0.776–1.331)	0.91
G T A	9 (0.8)	6 (0.9)	–	–
G T G	261 (22.2)	130 (19.1)	1.178 (0.930–1.492)	0.17

*p*, Fisher’s test; *p* considered as significant was bold.

**Table 4:** The disease activity and laboratory parameters in relations to *RORc2* rs9826 A/G; recessive model.

Parameter	AA + AG		GG		*p* *
	*N*	Median (IQR)	*N*	Median (IQR)	
Age (years)	470	56 (50–65)	63	58 (51–65)	0.52
Disease duration (years)	428	10 (4.5–15)	63	12 (7–20)	**0.02**
Larsen	469	3 (3–3)	64	3 (2–3)	0.66
Number of tender joints	262	7 (3–12)	42	6.5 (4–12)	0.85
Number of swollen joints	262	3 (1–7)	42	3 (0–7)	0.53
ESR (mm/h)	466	30 (17–50)	63	30 (17–47)	0.90
CRP (mg/L)	264	14.5 (6.7–35.5)	42	8.2 (3.2–15)	0.001
Hemoglobin (g/dL)	264	12.6 (11.5–13.6)	42	12.8 (12.1–13.3)	0.75
VAS (mm)	258	53 (32–71)	42	38.5 (23–60)	**0.01**
DAS 28-CRP	259	5.1 (3.9–5.9)	42	4.7 (3.6–5.8)	0.23
HAQ	248	1.5 (1.0–2.0)	38	1.1 (0.8–2.0)	0.14
PLT (×10^3^/mm^3^)	263	314 (255–387)	42	305 (237–366)	0.35
Creatinine	263	0.7 (0.6–0.8)	42	0.7 (0.6–0.9)	**0.02**
	**AA + AG**		**GG**		*p* **
	***N***	***n* (%)**	***N***	***n* (%)**	
Women	485	423 (87%)	65	62 (95%)	0.09
RF presence	464	321 (69%)	63	44 (70%)	0.92
Anti-CCP presence	266	220 (83%)	42	30 (71%)	0.13

IQR, interquartile range; *p* *, Mann–Whitney *U* test; *p* **, χ^2^ test or χ^2^ test with Yates’ correction; *p* < 0.003 was considered significant (according to Bonferroni correction); *N*, number of patients with clinical information; *n*, number of patients with positive information; *p* considered as significant was bold.

**Table 5:** The disease activity and laboratory parameters in relations to RORc4 rs9017 G/A; dominant model.

Parameter	GG		GA + AA		*p* *
	*N*	Median (IQR)	*N*	Median (IQR)	
Age (years)	61	57 (51–64)	432	55 (50–65)	0.72
Disease duration (years)	62	11.5 (7–18)	427	10 (5–15)	**0.04**
Larsen	62	3 (2–3)	431	3 (3–3)	0.53
Number of tender joints	38	6 (2–11)	227	8 (4–14)	0.20
Number of swollen joints	38	3 (0–5)	227	3 (1–7)	0.40
ESR (mm/h)	61	30 (17–45)	429	30 (19–50)	0.45
CRP (mg/L)	38	6 (3–13)	229	12 (6–30)	0.0005
Hemoglobin (g/dL)	38	12.9 (12.2–13.4)	229	12.6 (11.5–13.5)	0.29
VAS (mm)	38	35 (18–52)	226	52 (31–70)	0.006
DAS 28-CRP	38	4.4 (3.4–5.3)	225	5.0 (3.9–5.9)	0.06
HAQ	35	1.0 (0.8–1.6)	210	1.5 (1.0–2.0)	0.07
PLT (×10^3^/mm^3^)	38	296 (237–337)	228	311.5 (253–387.5)	0.17
Creatinine	38	0.7 (0.6–0.9)	229	0.7 (0.6–0.8)	**0.02**
	**GG**		**GA + AA**		*p* **
	***N***	***n* (%)**	***N***	***n* (%)**	
Women	63	60 (95%)	438	382 (87%)	0.10
RF presence	61	41 (67%)	431	293 (68%)	0.90
Anti-CCP presence	38	26 (68%)	230	182 (79%)	0.21

IQR, interquartile range; *p* *, Mann–Whitney *U* test; *p* **, χ^2^ test; *p* < 0.003 was considered significant (according to Bonferroni correction); *N*, number of patients with clinical information; *n*, number of patients with positive information; *p* considered as significant was bold.

**Table 6:** The disease activity and laboratory parameters (RA phenotype) in relations to RORc level.

Parameter	Protein Level			Protein Level			*p*
	Parameter Group I	*N*	Median (IQR)	Parameter Group II	*N*	Median (IQR)	
Age	age ≥ 56	142	2.22 (1.40–3.96)	age < 56	128	2.03 (1.29–3.62)	0.37
Sex	women	258	2.17 (1.37–3.82)	men	19	2.86 (1.26–4.64)	0.92
Disease duration	≥10	152	2.05 (1.38–3.73)	<10	114	2.28 (1.36–3.82)	0.83
Number of tender joints	≥7	156	2.28 (1.42–4.22)	<7	111	2.00 (1.16–3.77)	0.30
Number of swollen joints	≥3	143	2.33 (1.48–4.44)	<3	124	1.98 (1.18–3.39)	0.04
RF	RF+	171	2.30 (1.45–3.86)	RF−	100	1.83 (1.28–3.60)	0.12
Anti-CCP	a-CCP+	210	2.28 (1.40–4.03)	a-CCP–	60	1.75 (1.25–3.32)	0.07
ESR	≥30	126	2.28 (1.49–4.53)	<30	144	2.05 (1.29–3.62)	0.11
CRP	≥13	124	2.35 (1.49–4.44)	<13	145	2.00 (1.23–3.44)	0.07
DAS-28	≥5.0	127	2.37 (1.45–4.26)	<5.0	138	1.94 (1.18–3.64)	0.08
HAQ	≥1.5	126	2.40 (1.49–4.14)	<1.5	121	1.87 (1.31–3.56)	0.049

*N*, number of patients; RF+/–, positive/negative patients; a-CCP+/–, positive/negative patients; CAD+/–, positive/negative patients; IQR, interquartile range; *p*, Mann–Whitney *U* test; *p* < 0.05 was considered significant.

**Table 7:** Variation in RORc expression levels in RA patients and control group in relation to *RORc2* gene polymorphisms.

Genotype	RA Group		Control Group		*p*
	*N*	Median (IQR)	*N*	Median (IQR)	
RORC 2 rs9826 (A/G)					
AA	101	2.27 (1.27–4.14)	124	1.84 (1.24–2.84)	0.02
AG	132	2.02 (1.40–3.41)	135	1.91 (1.40–2.97)	0.35
GG	42	2.21 (1.51–4.56)	33	2.55 (1.64–3.92)	0.78
RORC 3 rs12045886 (T/C)					
TT	148	2.21 (1.42–3.82)	164	1.98 (1.25–3.16)	0.09
TC	108	2.49 (1.38–4.49)	116	1.92 (1.44–2.78)	0.04
CC	17	1.70 (1.33–2.19)	15	1.79 (1.10–2.46)	0.88
RORC 4 rs9017 (G/A)					
GG	39	2.21 (1.44–4.31)	37	2.33 (1.64–3.37)	0.36
GA	133	2.07 (1.44–3.64)	137	1.92 (1.41–2.97)	0.27
AA	104	2.28 (1.27–4.09)	121	1.80 (1.24–2.65)	0.01

IQR, interquartile range; *p*, Mann–Whitney *U* test; *p* < 0.05 was considered significant.

**Table 8:** Demographic and clinical characteristics of rheumatoid arthritis (RA) patients.

Characteristics	RA Patients	
	*N* *	Median (IQR)
Age (years)	557	56 (50–65)
Disease duration (years)	494	10 (5–16)
Larsen	538	3 (3–3)
Number of swollen joints	308	3 (1–7)
Number of tender joints	308	7 (3–12)
ESR (mm/h)	535	30 (17–50)
CRP (mg/L)	311	13 (6–32)
Hemoglobin (g/dL)	311	12.7 (11.6–13.5)
VAS (mm)	304	52 (32–70)
DAS 28-CRP	305	5.0 (3.9–5.9)
HAQ	291	1.5 (1.0–2.0)
PLT (×10^3^/mm^3^)	311	311 (254–383)
Creatinine	310	0.7 (0.6–0.8)
	*N* *	*n* ** (%)
Women	557	491 (88%)
RF presence	531	367 (69%)
anti-CCP presence	313	253 (81%)
Morning stiffness	335	262 (78%)

* *N*, number of patients with clinical information; ** *n*, number of patients with positive clinical manifestation, IQR—interquartile range ESR, erythrocyte sedimentation ratio; CRP, C-reactive protein; VAS, visual analogue scale (range 0–100); DAS-28, disease activity score for 28 joints; HAQ, Health Assessment Questionnaires (range 0–3); PLT, platelet; RF, rheumatoid factor; anti-CCP, anti-CCP antibodies.

## Supplementary Materials

Supplementary materials can be found at http://www.mdpi.com/1422-0067/17/4/488/s1.
