# Supplementary material for: RORC2 Genetic Variants and Serum Levels in Patients with Rheumatoid Arthritis
Source: Int J Mol Sci. 2016 Apr 1;17(4):488. doi: 10.3390/ijms17040488 (PMC4848944; doi:10.3390/ijms17040488)
Supplement: Supplementary file 1 [file ijms-17-00488-s001.pdf]

Supplementary Materials: RORC2 Genetic Variants and Serum Levels in Patients with Rheumatoid Arthritis

Agnieszka Paradowska-Gorycka, Barbara Stypinska, Andrzej Pawlik, Katarzyna Romanowska-Prochnicka, Ewa Haladyj, Malgorzata Manczak and Marzena Olesinska

Table S1. The disease activity and laboratory parameters in relations to *RORc2* rs9826 A/G; dominant model.

Parameter	AA		AG + GG		*p* *
	N	Median (IQR)	*N*	Median (IQR)	
Age (years)	204	56 (50–65)	329	56 (50–64)	0.84
Disease duration (years)	187	9 (5–15)	304	10 (5–17)	0.09
Larsen	204	3 (3–4)	329	3 (3–3)	0.73
Number of tender joints	110	8 (4–11)	194	7 (2–14)	0.69
Number of swollen joints	110	3 (1–6)	194	3 (1–8)	0.43
ESR (mm/h)	203	27 (15–45)	326	31 (18–50)	0.06
CRP (mg/L)	113	14 (6–24)	193	12 (6–30)	0.09
Hemoglobin (g/dL)	113	12.6 (11.7–13.7)	193	12.7 (11.6–13.3)	0.70
VAS (mm)	110	53 (32–70)	190	50.5 (30–71)	0.25
DAS 28-CRP	109	5.1 (4.2–5.8)	192	5.0 (3.8–6.0)	0.62
HAQ	107	1.4 (0.8–2.0)	179	1.5 (1.0–2.0)	0.34
PLT ($\times 10^3$/mm^3)	111	314 (246–390)	194	311.5 (255–379)	0.60
Creatinine	113	0.7 (0.6–0.8)	192	0.7 (0.6–0.8)	0.99
	**AA**		**AG + GG**		***p* ****
	N	*n* (%)	*N*	*n* (%)	
Women	212	186 (88%)	338	299 (89%)	0.80
RF presence	202	144 (71%)	325	221 (68%)	0.43
Anti-CCP presence	114	93 (82%)	194	157 (81%)	0.89

IQR—interquartile range; *p* *—Mann–Whitney *U* test; *p* **—$\chi 2$ test; $p < 0.003$ was considered significant (according to Bonferroni correction); *N*—number of patients with clinical information.

Table S2. The disease activity and laboratory parameters in relations to *RORc2* rs12045886 T/C; dominant model.

Parameter	TT		TC + CC		p *
	N	**Median (IQR)**	***N***	**Median (IQR)**	
Age (years)	261	56 (50–65)	229	56 (50–63)	0.41
Disease duration (years)	258	10 (5–16)	228	10 (5–16)	0.98
Larsen	261	3 (3–4)	229	3 (3–3)	0.28
Number of tender joints	143	8 (4–13)	119	8 (2–14)	0.95
Number of swollen joints	143	3 (1–6)	119	3 (0–7)	0.82
ESR (mm/h)	258	30 (17–50)	229	30 (19–50)	0.39
CRP (mg/L)	143	12 (5–32)	121	11.2 (5–21)	0.46
Hemoglobin (g/dL)	144	12.6 (11.6–13.5)	120	12.8 (11.7–13.6)	0.96
VAS (mm)	141	50 (34–67)	120	49.5 (28–68)	0.49
DAS 28-CRP	140	4.9 (4.0–5.9)	120	5.0 (3.7–5.9)	0.96
HAQ	130	1.4 (0.8–2.0)	112	1.5 (1.0–1.9)	0.83
PLT ($\times 10^3$/mm^3)	142	318.5 (265–386)	121	289 (234–370)	0.07
Creatinine	144	0.7 (0.6–0.8)	120	0.7 (0.6–0.8)	0.61
	**TT**		**TC + CC**		p **
	N	***n* (%)**	***N***	***n* (%)**	
Women	263	222 (84%)	235	217 (92%)	**0.006**
RF presence	260	179 (69%)	229	156 (68%)	0.86
Anti-CCP presence	143	112 (78%)	122	95 (78%)	0.93

IQR—interquartile range; p *—Mann–Whitney *U* test; p **—$\chi 2$ test; $p < 0.003$ was considered significant (according to Bonferroni correction); *N*—number of patients with clinical information; *p* considered as significant was bold.

Table S3. The disease activity and laboratory parameters in relations to *RORc2* rs12045886 T/C; recessive model.

Parameter	TT + TC		CC		*p* *
	N	**Median (IQR)**	***N***	**Median (IQR)**	
Age (years)	464	56 (50–65)	26	53 (50–61)	0.76
Disease duration (years)	459	10 (5–15)	27	11 (7–17)	0.18
Larsen	463	3 (3–3)	27	3 (3–4)	0.40
Number of tender joints	246	8 (4–13)	16	5 (2–10.5)	0.26
Number of swollen joints	246	3 (1–6)	16	2 (0–7.5)	0.43
ESR (mm/h)	460	30 (19–50)	27	30 (14–43)	0.35
CRP (mg/L)	247	12 (5–29.6)	17	10 (5–13)	0.34
Hemoglobin (g/dL)	247	12.7 (11.6–13.5)	17	12.7 (11.8–14.0)	0.65
VAS (mm)	244	50 (30–65)	17	51 (23–71)	0.59
DAS 28-CRP	243	5.0 (3.8–5.9)	17	4.6 (3.0–5.6)	0.29
HAQ	227	1.5 (1.0–2.0)	15	1.0 (0.4–1.3)	**0.02**
PLT (×10^3/mm^3)	246	308 (246–379)	17	316 (273–393)	0.87
Creatinine	247	0.7 (0.6–0.8)	17	0.7 (0.6–0.8)	0.65
	**TT + TC**		**CC**		***p*** **
	N	***n*** **(%)**	***N***	***n*** **(%)**	
Women	471	414 (88%)	27	25 (93%)	0.67
RF presence	462	317 (69%)	27	18 (67%)	0.99
Anti-CCP presence	248	195 (79%)	17	12 (71%)	0.64

IQR—interquartile range; *p* *—Mann–Whitney *U* test; *p* **—χ2 test or χ2 test with Yates' correction; $p < 0.003$ was considered significant (according to Bonferroni correction); *N*—number of patients with clinical information; *p* considered as significant was bold.

Table S4. The disease activity and laboratory parameters in relations to *RORc2* rs9017 G/A; recessive model.

Parameter	GG + GA		AA		p *
	N	**Median (IQR)**	***N***	**Median (IQR)**	
Age (years)	307	56 (50–64)	186	55.5 (50–65)	0.94
Disease duration (years)	306	10 (5–16)	183	9 (5–15)	0.18
Larsen	307	3 (3–3)	186	3 (3–4)	0.98
Number of tender joints	166	8 (2–14)	99	8 (5–12)	0.78
Number of swollen joints	166	3 (1–7)	99	2 (1–5)	0.33
ESR (mm/h)	305	32 (19–50)	185	28 (16–47)	**0.05**
CRP (mg/L)	166	10 (5–21)	101	13 (6–39)	**0.04**
Hemoglobin (g/dL)	165	12.8 (11.7–13.3)	102	12.5 (11.5–13.8)	0.80
VAS (mm)	165	50 (30–67)	99	52.9 (32–69)	0.25
DAS 28-CRP	165	5.0 (3.6–5.9)	98	5.0 (4.1–5.8)	0.58
HAQ	150	1.5 (1.0–2.0)	95	1.4 (0.8–2.0)	0.38
PLT ($\times 10^3$/mm^3)	166	309.5 (255–366)	100	306 (244.5–391)	0.76
Creatinine	165	0.7 (0.6–0.8)	102	0.7 (0.6–0.8)	0.97
	**GG + GA**		**AA**		p **
	N	***n* (%)**	***N***	***n* (%)**	
Women	312	277 (89%)	189	165 (87%)	0.62
RF presence	305	204 (67%)	187	130 (70%)	0.54
Anti-CCP presence	166	130 (78%)	102	78 (77%)	0.72

IQR—interquartile range; p *—Mann–Whitney *U* test; p **—$\chi 2$ test; $p < 0.003$ was considered significant (according to Bonferroni correction); ***N***—number of patients with clinical information; p considered as significant was bold.

Table S5. RORc2 protein level in RA patients and healthy subjects.

Parameter	RA Group		Control Group		p *
	N	**Median (IQR)**	***N***	**Median (IQR)**	
Protein Level (ng/mL)	278	2.2 (1.4–3.8)	295	1.9 (1.4–3.0)	**0.015**

p *—Mann–Whitney *U* test, RA *vs.* controls; $p < 0.05$ was considered significant; p considered as significant was bold.
